# Supplementary material for: Women's use of intimate apparel as subtle sexual signals in committed, heterosexual relationships
Source: PLoS One. 2020 Mar 13;15(3):e0230112. doi: 10.1371/journal.pone.0230112 (PMC7069627; doi:10.1371/journal.pone.0230112)
Supplement: Attachment — Submitted filename: Response to Reviewers.docx [file pone.0230112.s001.docx]

January 22, 2020

Dr. Amir H. Pakpour

Academic Editor, *PLOS* *ONE*

Dear Dr. Pakpour,

Thank you for the editorial response inviting us to revise our manuscript (PONE-D-19-31911: Women’s use of intimate apparel as subtle sexual signals in committed, heterosexual relationships) submitted previously to *PLOS ONE*. Per your suggestion, Lyndsey Craig has created an account for protocols.io to enhance reproducibility. Below, we itemize our responses to editor and reviewer comments. We appreciate the efficiency of this review process as well as the editor and reviewer’s comments helping craft a stronger manuscript.

Sincerely,

Lyndsey Craig and Peter Gray

**Editor Comments:**

**“While the topic is interesting but it’s clinical implication is not well defined. The authors need to clarify this in the manuscript throughout.”**

We thank the editor for their interest in this topic. To address the issue of clinical implication, we have included a paragraph under the Limitations and Future Research section (lines 539-551) to illustrate possible clinical significance of women’s use of intimate apparel.

**“Please ensure that your manuscript meets PLOS ONE’s style requirements, including those for file naming.”**

Per the editor’s comment, we have gone through the entire document to ensure it meets all style requirements of PLOS ONE including minor edits to the Title Page, elimination of Keywords section, and complete revision of tables and figures. We have also run the figures through the PACE digital diagnostic tool after reconfiguration. To the best of our knowledge, the manuscript now meets all style requirements.

**Reviewer Comments:**

**“Introduction: Explanation regarding the prices (line 133-138) does not seem to be necessary.”**

The reviewer’s comment is well-taken, though we still believe it important to emphasize the substantial cost of intimate apparel. Therefore, we removed lines 133-138 and added a simpler phrase to the sentence on line 131: “Although a more than 30 billion U.S. dollar market worldwide…”

**“Methods: psychometric properties of measures is not explained. linear regression has some assumptions. a brief explanation of how these assumptions were checked on this data might be useful for readers.”**

We agree with this comment and have added notes of reliability and validity to all relevant measures and made the methods section more transparent, such as separating the Intimate Apparel section to two sections: Attitudes and Use. We also created a separate section for the three Contexts. In the Statistical Analysis section, we specified how we measured *intent* and *initiation* (line 306) and provided a brief explanation about how we checked the assumptions of normality against using our statistical tests (lines 307-309).

**“Discussion: In this section main findings are explained but in some parts the findings are repeated. referring to tables is not appropriate for this part. the discussion should be enriched by adding more comparison with previous literature. It is seems better to add some explanation regarding the clinical implication of present findings.”**

This comment highlights flaws in the construction of the Discussion section. So, we have completely revised the Discussion section to eliminate redundancy and provide a logical flow of information. We have also removed any references to tables and have better incorporated previous literature.

As we mentioned in the editor comments, to address the issue of clinical implication, we have included a paragraph under the Limitations and Future Research section (lines 539-551) to illustrate possible clinical significance of women’s use of intimate apparel.
